# Progressive multifocal leukoencephalopathy — a diagnostic guide for the clinical neurologist

**DOI:** 10.3389/fneur.2026.1692522

**Published:** 2026-03-31

**Authors:** Dan Tong Jia

**Affiliations:** Department of Neurology, Northwestern University, Chicago, IL, United States

**Keywords:** clinical mimickers, diagnostic criteria, diagnostic delay, emerging biomarkers, granule cell neuronopathy, neuroimaging, neuroradiology, PML - progressive multifocal leukoencephalopathy

## Abstract

Progressive multifocal leukoencephalopathy (PML) is a rare, often fatal demyelinating disease of the central nervous system caused by reactivation of latent JC virus in immunocompromised individuals. Despite advances in understanding its pathogenesis, PML remains a diagnostic challenge due to its diverse clinical manifestations, radiographic overlap with other disorders, low incidence and subsequent lack of clinical familiarity. Diagnostic delays, often extending months after symptom onset, are common and contribute to poor outcomes. This review outlines the key clinical features, risk factors, diagnostic and neuroradiographic findings of PML to aid practicing neurologists in timely recognition and expedited diagnosis. We review emerging biomarkers and advanced diagnostic tools to identify PML. We also compare PML with its common mimics to underscore diagnostic pitfalls and how to overcome them. Novel and experimental therapies are beyond the scope of this article; instead, we focus on equipping neurologists with a framework grounded in patient data and clinical experience to establish timely diagnoses of PML.

## Introduction

Progressive multifocal leukoencephalopathy (PML) is a devastating demyelinating disease of the central nervous system (CNS) caused by human polyoma John Cunningham virus (JCV). JCV asymptomatically infects 60% of the general population, and remains latent in the kidneys, bone marrow, and lymphoid tissues. In immunocompromised patients JCV can reactivate and undergo mutations creating neurotropic variants that infect glial cells. These variants can spread to the brain and infect oligodendrocytes causing CNS demyelination (1). Following the initial description of PML in a patient with chronic lymphoid leukemia in 1958, the high rates of PML observed in HIV patients during AIDs pandemic, multiple sclerosis (MS) patients after natalizumab use, and transplant patients on immunosuppression highlight the heightened PML risk in immunocompromised populations (2–5). PML is characterized by progressive neurologic decline and high mortality, ranging from 30–90% depending on underlying sources of immunosuppression, with lower rates in HIV-associated cases and higher in hematologic malignancies (6, 7). Although no FDA approved therapeutics are available for PML, rapid reversal of underlying immunocompromised state, such as initiation of antiretroviral therapies (ART) in HIV-associated PML or discontinuation of natalizumab in MS patients, has been associated with better functional outcomes (7–10). As such, early clinical recognition and rapid diagnostic evaluation of PML are crucial.

Despite our progress in understanding PML in the past half century, its clinical diagnosis remains a challenge for the practicing neurologist given its rare incidence, diverse clinical manifestations, and neuroradiographic similarities to other diseases. On average, PML patients experience a diagnostic delay of 1–3 months from initial symptom onset, with 10% of patients delayed over 1 year (6, 11). These diagnostic delays are attributable to workup and treatment of mimicking etiologies and errors in diagnostic heuristics. As such, practicing neurologists must be familiar with the clinical manifestations and diagnostic pearls of PML. Here, we will highlight key features of PML by reviewing symptomatology, risk factors, diagnostic testing, and neuroradiographic findings that can help distinguish PML from common mimickers. We will also review emerging serum and cerebrospinal fluid (CSF) biomarkers that can support the diagnosis. While no effective therapies are currently available for treatment of PML, emerging immunomodulatory strategies such as immune checkpoint inhibitors and virus-specific T-cell transfers have shown promise. However, treatment of PML lies outside the scope of this review.

## Clinical presentation of PML

JCV primarily infects oligodendrocytes so PML affects the subcortical, periventricular, and cerebellar white matter, although deep gray matter structures such as the thalamus can be involved as well. Patients usually present with progressive, multifocal, subacute neurologic deficits that depend on the affected supratentorial or infratentorial structures; speech and coordination difficulties, cortical visual deficits, cognitive changes, apraxia, and motor and sensory deficits are frequently reported (12, 13). Seizures can occur in up to 40% of PML patients, especially those with juxtacortical lesions (8, 14, 15). Despite rare histopathologic reports of spinal cord involvement, the optic nerves and spinal cord are typically spared symptomatically and radiographically in PML patients (16, 17). This contrasts other classic demyelinating diseases like MS, neuromyelitis optic spectrum disease (NMOSD), and myelin oligodendrocyte associated disease (MOGAD). Subacute progression of existing neurologic symptoms and additional focal deficits over several days to weeks should raise clinical concern for PML.

Rarely, JCV can infect granule cells of the cerebellum and cause a clinically distinct disease called granule cell neuronopathy (GCN). GCN was initially described as subacute progressive cerebellar ataxia due to isolated cerebellar granule cell layer atrophy, usually in the absence of supratentorial lesions and symptoms (18). Recent reports reveal heterogenous lesional burden suggesting overlap with PML in some GCN patients (19).

## Epidemiology and predisposing risk profiles

PML is a rare neurologic disease, affecting 0.11 per 100,000 individuals annually per year, but there were two notable historic peaks in incidence (20, 21). At the height of the AIDS epidemic, PML affected approximately 5% of individuals living with HIV; with the advent of ART, this incidence has declined more than 10-fold (2, 13). Similarly, long-term outcome data in MS patients treated with natalizumab, a high-efficacy disease modifying therapy approved in 2005, have demonstrated a heightened incidence of PML with 20 cases for every 1,000 patients (22, 23). Several risk stratifying measures, such as using JC virus antibody index to reduce natalizumab exposure in high-risk patients, have significantly reduced the rate of PML in natalizumab treated patients (24). A recent, large population cohort of PML patient from France demonstrated the most common predisposing risk factor remains HIV infection (44%), followed by hematologic malignancies (22%), chronic inflammatory diseases (20%), solid organ transplantation (4%), solid cancers (4%) and primary immune deficiencies (2%) (21). These vulnerable patient populations highlight the critical need for practicing neurologists to recognize the risk profile for PML and promptly evaluate predisposed individuals. As PML does not manifest uniformly across all immunocompromised populations, we will review the high yield at-risk patient populations below:

### HIV

PML is strongly associated with low CD4 + count in individuals living with HIV. Patients with CD4 + counts under 200 cells/μL have a 45 times higher relative risk of PML compared to those with CD4 + counts over 200. Severe CD4 + lymphopenia under 50 is associated with a higher mortality rate, which is likely attributed to the increased risk of PML-immune reconstitution inflammatory syndrome (PML-IRIS). PML-IRIS is characterized by paradoxical symptomatic and radiographic worsening following ART initiation. PML-IRIS can occur from weeks to months after ART initiation or adjustment, and is associated with higher morbidity and mortality (25). It remains a difficult challenge to clinically differentiate PML-IRIS from PML progression, but we will review characteristic radiographic findings of PML-IRIS in a later section.

Notably, low CD4 + count is not a prerequisite for PML in HIV patients. The synergistic coinfection of HIV and latent JCV can promote JCV reactivation and viral replication, irrespective of CD4 + counts (26). The absence of overt immunosuppression is a common diagnostic heuristic that can mislead clinicians and contribute to the significant diagnostic delay of PML in HIV patients with CD4 + counts over 200 (11).

### Natalizumab treatment

Treatment with natalizumab, a monoclonal antibody that blocks immune cell migration into the CNS, increases risk of PML. Natalizumab-associated PML was first reported in clinical trials for its use in relapsing remitting multiple sclerosis (RRMS) and Crohn’s Disease (27, 28). Duration between natalizumab treatment initiation and PML symptom onset was 28 month on average and risk for PML progressively increases up to 3 years after treatment initiation (29). Bloomberg and colleagues found presence of JCV antibodies, history of prior immunosuppression, and natalizumab treatment for 25–48 weeks as independent risk factors for developing natalizumab-associated PML (30). Serum JCV antibody index less than 0.9 in patients without prior immunosuppression is considered low risk for PML on natalizumab treatment, however some experts suggest more conservative low-risk index of 0.6 or less (31–33).

Plasma exchange was historically recommended in natalizumab-associated PML patients to rapidly remove the offending antibody and restore immunosurveillance (9, 34). However, recent studies suggest plasma exchange may not improve survival or reduce poor neurologic outcomes in this patient population. This likely stems from high rates of PML-IRIS following plasma exchange, which in turn is associated with worse neurologic outcomes (10, 35, 36).

### Additional immunosuppressive medications

PML has been observed in other therapies that impede lymphocyte migration, such as efalizumab for psoriasis which led to its withdrawal from markets worldwide, and sphingosine 1-phosphate receptor modulators for MS (37, 38). Several additional classes of MS disease-modifying therapies are also associated with PML, albeit at lower rates, including fumaric acid derivatives, such as dimethyl fumarate, B-cell depleting therapies such as rituximab, ofatumumab, ocrelizumab, and the anti-CD52 monoclonal antibody aletumumuab (39, 40). Additionally, chemotherapeutic agents have been reported to cause PML, likely due to their profound immunosuppressive effects that permit JCV reactivation, and these include fludarabine, brentuximab, azathioprine, and cyclophosphamide (41).

PML is a rare complication of solid organ transplantation due to required immunosuppression, with an incidence of 1.24 cases per 1,000 patients per year (42, 43). Cases of PML have also been reported in patients receiving both autologous and allogenic stem cell transplantation (SCT) for hematologic diseases (44, 45). Chronic immunosuppression in allogenic SCT patients to prevent graft-versus-host disease further predispose these patients to PML (46). Limited management options are available as reducing immunosuppression incurs significant risk of rejection and worsening graft-versus-host disease, with only one case report of successful treatment with immunotherapy in an allogenic SCT patient found in the literature (47).

Risk of PML in hematologic malignancies is compounded by the inherent immunocompromise state, the use of cytotoxic chemotherapies such as cyclophosphamide, and immune ablation prior to SCT (44, 48). Certain monoclonal therapies, such as anti-CD20 rituximab for lymphomas and anti-CD30 brentuximab for anaplastic large cell and Hodgkin’s lymphoma, have been associated with PML (49, 50). PML is more frequently reported in lymphoid than myeloid neoplasms (51). Interestingly, PML has rarely been reported in patients with treatment-naïve chronic lymphocytic leukemia, highlighting the potential inherent risk of PML associated with hematologic malignancy itself (48).

### Underlying systemic diseases

Rheumatologic diseases like sarcoidosis, granulomatous polyangiitis, rheumatoid arthritis and systemic lupus erythematosus (SLE) have been associated with PML in retrospective case series, but many of these cases received concurrent immunosuppressive therapy that likely contributed to the risk (6, 52). Interesting, SLE is the predominant rheumatologic disease associated with PML, up to 65% in one retrospective series of PML patients, with an elevated prevalence of 13–27 PML cases per 100,000 patients with SLE (53, 54). As these SLE patients were exposed to minimal iatrogenic immunosuppression, authors have proposed that SLE may confer unique, currently unelucidated, risk for developing PML.

The risk for PML in systemic sarcoidosis is multifactorial, including intrinsic immune dysregulation, CD4 + lymphopenia, and the use of chronic immunosuppressive medications (55, 56). A recent study suggests that sarcoidosis is associated with PML even for patients who are not on immunosuppression and only exhibit mild to moderate CD4 + lymphopenia (57). Interestingly, simultaneous diagnosis of PML and systemic sarcoidosis was common in this study. Clinicians must exercise caution to avoid misdiagnosing PML as neurosarcoidosis in patients with systemic sarcoidosis as it can lead to excessive diagnostic delays and detrimental escalation of immunosuppression (58).

Idiopathic CD4 + lymphopenia (ICL) is rare but a significant risk factor for PML. ICL is defined as absolute CD4 + count less than 300 cells/μL without offending immunosuppressive therapies or immunodeficiency and is a rare but well documented cause of PML in 28 case studies (59). One case of PML in a patient with profound lymphopenia exhibited mutations in GATA2 and CDH7 genes implicated in T and B lymphocyte production, which highlights genetic contributions to PML risk. Clinical vigilance for ICL and genetic immunodeficiencies, by inquiring about past opportunistic infections, checking T and B lymphocytes counts, and appropriate vaccine antibody titers can identify subtle immunocompromise, shift risk stratification for PML, and prevent diagnostic delays up to 2 years in one large case series (6).

## Diagnostic testing in PML

The PML diagnostic criteria was established by Berger and colleagues in 2013 and categorizes diagnostic certainty to definitive, probable, and possible PML based on presence of compatible clinical features, neuroradiographic findings, and CSF JCV PCR positivity (60). Definitive PML is reached when all three parameters (clinical, radiographic, and JCV PCR positivity) are present. A diagnosis of probable PML can be made when CSF JCV PCR is positive and either compatible clinical or neuroradiographic features (but not both) are present. Possible PML is reserved for cases with both compatible clinical and radiographic features, but negative CSF JCV PCR. Rare instances where patients may have low CSF positive JCV viral loads without any clinicoradiographic findings are classified as possible PML as it may reflect normal biology of JCV or a preclinical state of PML (61). These patients should be monitored regularly for progression of symptoms or lesions on magnetic resonance imaging (MRI). Additionally, a definite PML diagnosis can be reached by brain tissue biopsy with presence of the histologic triad of multifocal demyelination, enlarged oligodendrocytes with swollen nuclei, and bizarre astrocytes, plus either immunohistochemistry (IHC) staining for SV40 highlighting intranuclear JCV inclusions in oligodendrocytes or positive tissue PCR for JCV DNA. Given potential challenges of neurosurgical availability, safe biopsy site, sampling error, and post-operative complications, brain biopsies are typically reserved for 25% of PML cases that initially have undetectable CSF JCV virus or when neoplasm remains a concern (11, 62).

CSF sampling is a crucial step in the diagnostic process for PML to detect presence of JCV, as well as ruling out mimicking etiologies. PML is associated with a relatively benign CSF profile. The average CSF white cell count was 5–7 cells/mm2 with lymphocytic predominance in 80% of patients, mildly elevated CSF protein to 60 mg/dL, with normal CSF glucose and lactate (2, 12, 63). In a subgroup analysis between etiologies of PML, HIV-associated PML patients had slightly higher CSF cell counts, protein, and lactate compared to natalizumab-associated PML (63).

CSF JCV PCR testing has improved significantly. Early assays detecting >200 viral copies/μL showed 40–75% sensitivity, whereas current ultrasensitive PCRs capable of detecting 10–20 copies/μL achieve 85% sensitivity on initial testing and 95% with repeat CSF sampling (64, 65). Compared to HIV-associated PML, natalizumab-associated PML typically exhibits lower or undetectable CSF JCV viral loads (66). Wijburg and colleagues found an association between PML lesional volume and CSF JCV viral loads, which suggest that false negatives are more likely with smaller lesions and underscores the value of repeat testing following lesional progression (67). In addition, a reconstituting or mildly compromised immune system, such as in patients with PML-IRIS or idiopathic CD4 + lymphopenia, may suppress viral replication and lead to falsely negative CSF PCR results (68). An elevated CSF JCV antibody index can indicate CNS JCV activity by demonstrating intrathecal antibody production against the virus; a useful tool in cases with low or undetectable CSF JCV viral loads (66, 69). A CSF antibody index greater than 1.5 may support possible PML cases when CSF JCV PCR is negative, but the assay is not widely available and should be interpreted with caution given lack of studies across larger patient cohorts.

Blood tests assist with PML risk stratification by screening for latent JCV and immunocompromised states. JCV latency is assessed with JCV seropositivity or antibody index, where values above 0.6–0.9 indicate increased JCV reactivation risk in patients treated with natalizumab (33). Notably, systemic JCV latency is common with antibodies present in 50–70% of the general population, and are not independently sufficient to establish a diagnosis of PML (70). Similarly, JCV DNA is detectable in peripheral blood in 20% and in urine in 33% of the general population. Both tests should be interpreted cautiously and in conjunction with the greater clinical picture (71, 72).

Evaluation of PML risk factors should begin with detailed medication history over prior 2 years and any personal or family history of unexplained or opportunistic infections. Comprehensive workup for immunodeficiency includes infectious causes (HIV, EBV), rheumatologic conditions (SLE, sarcoidosis, systemic vasculitis), systemic neoplasms (multiple myeloma, lymphoma, leukemia, metastatic cancers), and neuroimmunologic conditions (MS, NMOSD, MOGAD). Screening total lymphocytes count, B and T cells counts, and immunoglobulins can reveal underlying immunodeficiencies and may trigger further genetic evaluation if no secondary causes are identified. While no blood test is diagnostic, results can support clinical suspicion for PML, re-orient diagnostic heuristics, and accelerate confirmatory CSF or brain biopsy evaluation for JCV.

## Neuroradiographic findings

PML is a demyelinating disease characterized by predominantly white matter lesions. On initial imaging, 70% of patients exhibit multifocal lesions and nearly half show infratentorial disease involvement (6). Brain MRI is the most sensitive imaging modality to evaluate lesions across multiple sequences. However, the radiographic overlap PML shares with vascular, demyelinating, and neoplastic mimickers often delay diagnosis and management. Below, we review classical appearance and distinguishing features of intracranial PML lesions across various imaging modalities.

On head computerized tomography (CT), PML appears as confluent hypodense lesions with minimal mass effect and no contrast enhancement. Correspondingly, MRI T1 pre-contrast sequences show hypointense to isointense lesions at disease onset that progress to markedly decreased signal intensity, reflecting severe demyelination and encephalomalacia (73). Early HIV-associated PML cohorts suggest that an overwhelming majority of lesions demonstrate no contrast enhancement on MRI T1 post-contrast sequences, with only 15% exhibiting faint and peripheral enhancement (2). Subsequent large, multi-etiologic PML cohorts found lesional enhancement in up to 25% of cases (6, 20). In a Swedish national PML cohort, lesional enhancement was observed in 6% of HIV-associated cases and 15% of iatrogenic/primary immunodeficiency cases. Surprisingly, their cohort had 7 PML patients that were previously healthy without immunosuppressive history and 3 of these patients exhibited contrast enhancing lesions on initial MRI (20). Collectively, emerging evidence suggests that contrast enhancement is more common than previously recognized and may be inversely correlated with degree of immunosuppression.

**Figure 1:**
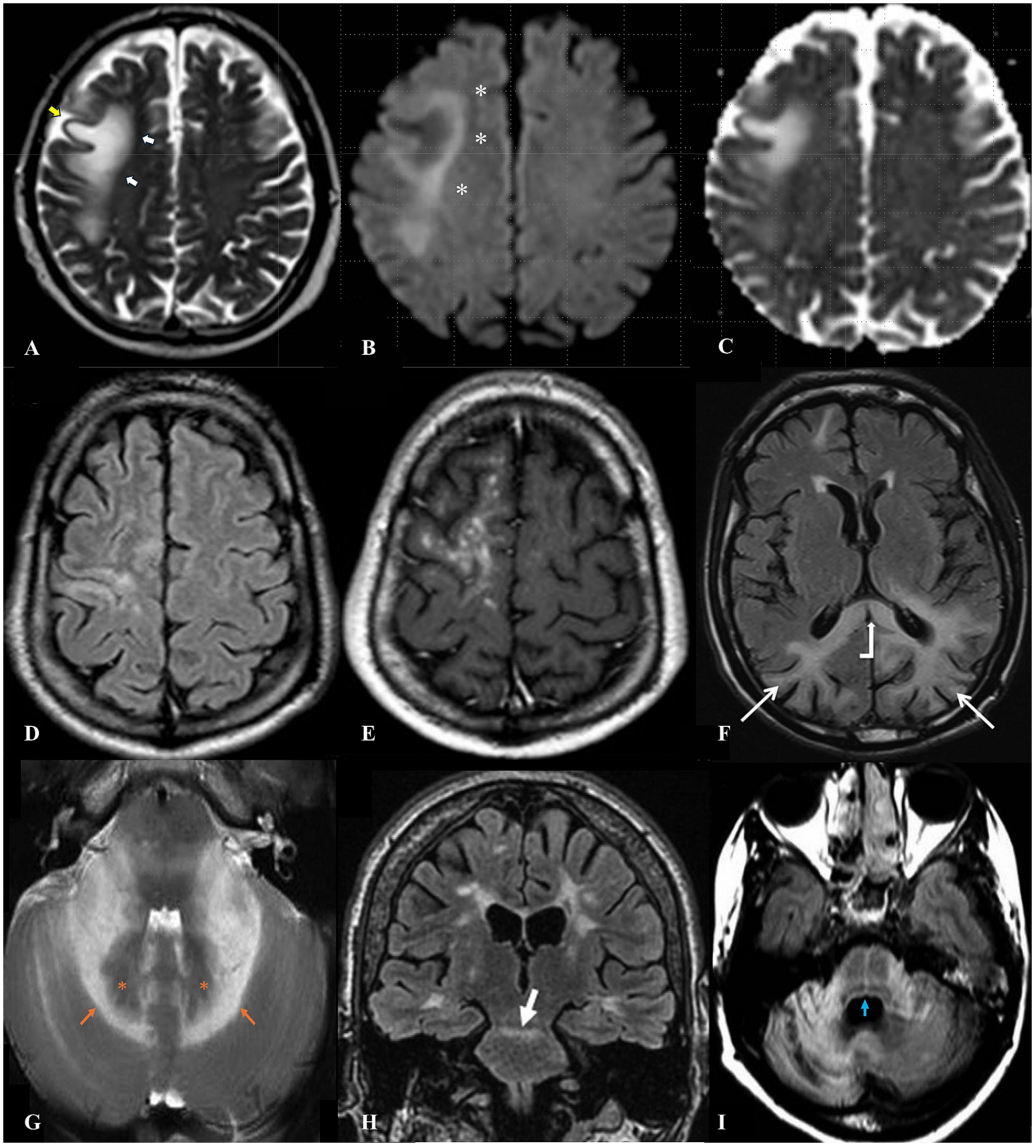
Brain MRI findings in PML. Leading edge diffusion restriction **(A–C)**: Classic PML lesion appearance on T2 sequence with sharp juxtacortical margin (**A**, yellow arrow) and poorly defined subcortical white matter margin (**A**, white arrows). Leading edge diffusion restriction on DWI B1000 sequence (**B**, white asterisks) along the subcortical margin with corresponding pseudo-normalization on ADC sequence **(C)**, corresponding to a zone of active demyelination and lesion expansion (73). Milky way sign **(D,E)**: Punctate lesions of T2-FLAIR hyperintensity **(D)** exhibiting contrast enhancement on T1-post-contrast **(E)** seen within or adjacent to PML lesions, corresponding to areas of active perivascular inflammation (111). Barbell sign **(F)**: Confluent occipital T2-FLAIR hyperintensities (**F**, white straight arrows) that extend across the splenium (**F**, white bent arrow), which appears atrophic and thin in size (84). Shrimp sign **(G)**: Bilateral or unilateral T2 hyperintensities (**G**, orange arrows) in the cerebellar white matter that spare and outline dentate nuclei (**G**, orange asterisks), forming a shrimp shape on axial sequences (80). Across the pons sign **(H)**: Rostral pontine T2-FLAIR hyperintense lesion (**H**, white arrow) that extends across the midline, seen on coronal plane (82). Hot cross bun sign **(I)**: Mid-pontine T2-FLAIR cruciform hyperintense lesion (**I**, blue arrow) that can also be seen in MSA, but rarely in vascular, infectious, inflammatory, or tumoral mimickers of PML (81).

On T2 FLAIR sequences, PML lesions appear confluently hyperintense with sharply delineated juxtacortical margins and ill-defined borders toward subcortical white matter (Figure 1A) (74). Diffuse-weighted imaging (DWI) often shows “leading-edge” hyperintensity/diffusion restriction along the subcortical periphery of the lesion, corresponding to active demyelination and lesion expansion (Figures 1B,C) (75, 76). Another common PML radiographic feature is the “milky way sign”, defined by punctate T2 hyperintense lesions (often with contrast enhancement), within or adjacent to large confluent T2 hyperintense lesions (Figures 1D,E). Studies postulate the milky way sign reflects perivascular inflammation and disease progression, as these punctate lesions typically lose contrast enhancement and coalesce with the expanding confluent lesion over time (77–79). Posterior fossa predominant PML is rare but can exhibit unique MRI signs. The “shrimp sign” depicts T2 hyperintense cerebellar lesions that surround, sharply demarcate and outline the uninvolved dentate nucleus (Figure 1G). “Across-the-pons” and “hot crossed buns” signs refer to the cruciform T2 lesions that span bilaterally across the pons (Figures 1H,I). The latter is commonly ascribed to multiple systems atrophy, but rarely reported in PML mimics and distinguishes PML from posterior fossa strokes and other demyelinating lesions (80–82). GCN classically appears with isolated marked cerebellar gray matter atrophy, but overlap with PML has been reported with concurrent supratentorial and infratentorial white matter lesions, including a GCN case with pontine hot cross bun sign (83). Lastly, the “barbell sign” describes confluent occipito-parietal T2-FLAIR lesions that extend across the splenium, but notably lack intralesional contrast enhancement or diffusion restriction, as well as evidence of splenial expansion (Figure 1F) (79, 84). Unlike splenium signal abnormalities seen in glioblastoma or metabolic encephalopathies, the lack of enhancement, diffusion restriction, and volume loss of the splenium in the barbell sign make it a relatively specific radiographic feature for PML.

PML-IRIS results from the pro-inflammatory state that emerges following removal or reversal of immunosuppression, such as with initiation of ART in HIV patients or plasma exchange in natalizumab-associated PML. Radiographically, previously confluent T2-FLAIR lesions develop new patchy or punctate contrast enhancement with lesional expansion. Severe cases may demonstrate marked lesional growth, mass effect, and, if untreated, midline shift and herniation (73, 85). PML-IRIS may be the initial presentation of PML in patients in whom the clinicoradiographic findings of PML are unmasked following initiation of ART in HIV (86). In such cases, there is a risk that atypical radiographic features may result in a delay in diagnosis. Therefore, the presence of enhancement or mass effect should not rule out the diagnosis of PML and the imaging should be interpreted within the entire clinical context.

Auxiliary imaging studies can provide additional diagnostic support for PML in appropriate clinical context. Magnetic resonance spectroscopy (MRS) evaluates metabolic composition of the selected lesional voxel. MRS of PML lesions demonstrates reduced N-acetylaspartate peak, elevated choline peak, and a prominent lactate peak compared to control voxels (73, 87). Positron emission tomography (PET), though rarely reported, shows low FDG uptake suggestive of hypometabolism in PML lesions (73, 88). This can be helpful to differentiate PML from hypermetabolic lesions like infections and neoplasm. Both imaging modalities are typically limited to academic tertiary centers and should be interpreted cautiously given paucity of data in large patient cohorts.

## Discussion

### Challenges in PML diagnosis

We have reviewed clinical manifestations and risk factors profiles that raise suspicion for PML and should prompt targeted evaluation. Despite these frameworks, diagnostic delay and misdiagnoses remain common and negatively affect outcomes. A retrospective study from a Boston neuroinfectious disease clinic reported a median diagnostic delay of 74 days from symptom onset to PML diagnosis (11). In the HIV + cases, a higher CD4 + count correlated with longer delay, likely reflecting the misconception that PML occurs only in advanced immunosuppression. Notably, 14 of 65 HIV-associated PML patients had CD4 + counts above 200 and most experienced over 3-month delays. Another report described a 2-year delay in a PML patient with idiopathic lymphopenia (6). As PML survival improves when expedited diagnoses occur within 1 month from symptom onset, thorough screening for PML risk factors should be conducted comprehensively and early in the clinical course.

Misdiagnoses occur in up to two-thirds of patients before reaching the correct PML diagnosis (11). In the national Swedish cohort, patients seen on non-neurologic services were almost four times more likely to experience PML misdiagnoses than patients evaluated by neurologists (20). Common misdiagnoses for PML patients included vascular lesions, other CNS infections, neurosarcoidosis, and neoplasms; thorough diagnostic evaluations of such entities further delayed PML diagnoses. This underscores the value of clinical familiarity and targeted suspicion for PML among practicing neurologists.

PML is frequently mistaken for vascular disease, including ischemic stroke or CNS vasculitis, due to focal neurologic deficits, new diffusion-restriction lesions, and progressive T2 hyperintense lesions on MRI (89, 90). Especially in HIV-associated PML, concurrent HIV-driven cerebrovascular changes such as accelerated atherosclerosis and high microvascular disease burden can further complicate the differential diagnoses (91). The subacute and progressive course of neurologic deficits in PML contrasts the abrupt onset and subsequent stabilization and recovery seen in ischemic strokes. Radiographically, PML exhibits leading-edge diffusion restriction along the subcortical periphery of the lesion, unlike the vascular territory-based diffusion restriction in ischemic strokes or the punctate multifocal diffusion restriction and concentric vessel wall enhancement seen in CNS vasculitis. Contrast enhancement can occur in both PML, PML-IRIS, or subacute infarcts. Screening for underlying immunocompromise for risk stratification and close symptomatic and radiographic followup will be crucial to avoid vascular misdiagnoses.

CNS neoplasms are another common mimic and share many clinical similarities with PML. Both PML and gliomas are characterized by subacute progressive neurologic decline. Seizures are more likely in neoplasms but also occur in PML patients (92). Radiographically, PML and low-grade gliomas can both present with T2 hyperintense lesions with little to no enhancement, although PML lesions tend to be multifocal and lack mass effect. High-grade gliomas are usually associated with avid enhancement, perilesional edema, and mass effect, which are uncommon in PML except in cases of PML-IRIS. The barbell sign helps differentiate PML from splenial GBM or NMOSD lesions by its lack of contrast enhancement and splenial atrophy rather than edema and expansion. MRS can support PML with a prominent lactate peak, while CNS neoplasms show markedly elevated choline peaks as part of the reversed Hunter’s angle pattern (93). PET scans can reveal hypometabolism in PML versus hypermetabolism in neoplasms, but PML-IRIS lesions can exhibit higher FDG uptake and should be interpreted with caution (94, 95). As PML and CNS neoplasm overlap in several key clinicoradiographic features, histopathology may be needed to establish a definitive diagnosis.

Distinguishing PML and new MS lesions, particularly in patients treated with natalizumab or other high-risk disease-modifying therapies, remains challenging as both can demonstrate leading-edge diffusion restriction and peripheral enhancement. MS lesions are typically ovoid and well-circumscribed, whereas PML can be more confluent on T2 sequences with less defined subcortical borders (96). The central vein sign and paramagnetic rim sign are emerging MRI biomarkers with relatively high specificity for MS and may be useful for distinguishing MS from PML (73); however, additional data is needed to support its use in this setting.

PML is commonly misdiagnosed as neurosarcoidosis in patients with systemic sarcoidosis, up to 80% in one case series (58). Cognitive heuristics to identify a unifying diagnosis can mislead clinicians when systemic workup for intracranial PML lesions lead to a diagnosis of systemic sarcoidosis. Furthermore, sarcoidosis-associated PML can exhibit contrast enhancement in 30% of cases which can further confuse clinicians (57). Identification of PML-specific patterns of contrast enhancement, such as milky way or shrimp sign, can be clinically helpful. The absence of cranial nerve involvement or pachy- and leptomeningeal enhancement, which are present in many patients with neurosarcoidosis, can also be an important clue. Progression of non-enhancing lesions also suggest PML over neurosarcoidosis. Initial CSF profiles can help distinguish between the two entities, where PML tends to have a relatively bland CSF profile compared to neurosarcoidosis, which is characterized by pleocytosis, elevated protein, and hypoglycorrhachia (97, 98). Confirmatory CSF tests for PML should be performed early in sarcoidosis patients to avoid diagnostic delays and harmful treatment escalations associated with misdiagnosis.

Table 1 outlines distinguishing clinicoradiographic features of PML compared to its common mimickers discussed above.

**Table 1:** Summary of distinguishing features of PML (green column) compared to its common clinical mimickers.

PML	Ischemic strokes	CNS vasculitis	CNS neoplasms	Multiple sclerosis	Neurosarcoidosis
Clinical presentation					
Subacute, progressive neurologic decline. Focal neurologic deficits with intact mental status initially Spares optic nerves and spinal cord Can present with seizures	Abrupt onset with focal deficits Subacute stabilization and recovery phase	Subacute, progressive neurologic decline Confusion and encephalopathy Headaches Can present with seizures	Subacute progressive deficits Commonly presents with seizures B symptoms	Relapsing–remitting symptoms in majority of cases Commonly involve optic nerves and spinal cord	Subacute, progressive deficits Can present with headaches, cranial neuropathies, parenchymal and myelopathic lesions.
Risk factors					
Immunocompromised state Immunosuppressive medications HIV co-infection	Coagulopathy Atrial Fibrillation Vasculopathic risk factors	Rheumatologic diseases Connective tissue disorders Para-infectious triggers Neurotropic infections	Genetic risk factors Prior radiation exposure Systemic metastatic neoplasms	Female Genetic risk factors Vitamin D deficiency EBV infection	Female African American descent Caucasian descent Systemic sarcoidosis diagnosis
Diagnostic workup					
Identification of immunocompromised state Serum JCV antibody, antibody index CSF JCV PCR, CSF JCV antibody index Brain biopsy: multifocal demyelination enlarged oligodendrocytes bizarre astrocytes SV40 positive staining/JCV PCR	Vasculopathic risk factor identification Centroembolic source identification Vessel imaging	MRI vessel wall imaging Diagnostic angiogram Skin biopsy	Serum cancer biomarkers Systemic imaging for neoplasm Brain biopsy	Clinical and radiographic satisfaction of McDonald’s Criteria CSF testing for oligoclonal bands, IgG index, and Kappa free light chains	Systemic sarcoidosis evaluation Neurosarcoidosis Consortium Consensus Group Diagnostic Criteria 2018 CSF with pleocytosis, elevated protein, and/or hypoglycorrhacia Brain biopsy with granulomas
Neuroimaging findings					
CT: confluent, hypodense lesions. MRI: T2 hyperintense, confluent white matter lesions with less defined borders; infrequent contrast enhancement Leading-edge diffusion restriction Milky way sign Shrimp sign Across-the-pons sign Hot crossed buns sign Barbell sign MRS: prominent lactate peak, elevated choline and reduced N-acetylaspartate peaks PET: relative hypometabolism within lesions	Lesions involve both grey and white matter with cytotoxic edema MRI: bright diffusion restriction that follow vascular territories. Contrast enhancement seen in subacute phase, usually in gyriform pattern.	MRI: progressive confluent T2 hyperintense lesions. Multifocal, punctate diffusion restriction lesions. Associated with GRE + MRI vessel wall imaging with concentric vessel wall enhancement	Juxtacortical lesions for metastatic lesions with adjacent vasogenic edema. ariable degree of contrast enhancement dependent on tumor. Diffusion restriction signal for highly cellular tumors like CNS lymphoma	MRI: ovoid, well-circumscribed white matter lesions in the periventricular, juxtacortical, brainstem, and spinal cord Central vein sign for periventricular lesions Paramagnetic rim sign for chronic demyelinating lesions	MRI: Intracranial parenchymal lesions are generally contrast enhancing, exhibit peri-lesional edema and can appear tumefactive. Multiple cranial neuritis, including optic neuritis. Dural thickening and pachymeningitis. Longitudinally extensive myelitis with trident sign.

PML, progressive multifocal leukoencephalopathy; JCV, John Cunningham virus; HIV, human immunodeficiency virus; CSF, cerebrospinal fluid; PCR, polymerase chain reaction; CT, computed tomography; MRI, magnetic resonance imaging; MRS, magnetic resonance spectroscopy; PET, positron emission tomography; GRE, gradient echo; EBV, Ebstein-Barr virus; IgG, immunoglobulin G.

### Recent advancements in PML diagnostics

Presence of CSF JCV is crucial for the definitive diagnosis of PML when deep lesions or access to neurosurgery preclude biopsy. Many reference laboratories have used ultrasensitive CSF PCR assays for increased sensitivity since the 2010s. However, persistently negative CSF ultrasensitive PCR results have been reported in patients with biopsy or autopsy confirmed PML. These false negative CSF results were attributed to low circulating viral target DNA copies, insufficient CSF sample volumes, transportation delays and unspecified limits of quantification and detection across reference laboratories (62, 66, 99–101). Novel molecular diagnostics, such as CSF metagenomic next generation sequencing (mNGS), improve sensitivity and specificity for detecting CNS infection and can identify pathogens in 20–40% of negative cases by conventional testing (102–104). In their 7-year experience of using CSF mNGS for CNS infections, Benoit and colleagues reported 11 positive reads for JCV, with 1 positive result on mNGS that provided additional information compared to conventional testing (102). CSF mNGS may aid evaluations patients with possible PML and repeatedly negative CSF ultrasensitive JCV PCRs. Future studies to directly compare sensitivities between mNGS and ultrasensitive PCR in suspected PML patients will clarify their clinical utility.

Additionally, serum and CSF biomarkers are under investigation to support clinical suspicion for PML. Neurofilament light chain (NfL) is released during axonal injury, can cross the blood brain barrier, and are elevated in demyelinating diseases. Natalizumab-treated MS patients who developed PML exhibited 8-10x higher serum NfL compared to control MS patients, both during remission and active relapses (105, 106). Elevated CSF IL-10 levels have also been reported in early natalizumab-associated PML cases compared to healthy controls (107, 108). IL-10 is an important anti-inflammatory cytokine to prevent excessive tissue damage, but pathologically elevated levels are associated with persistent viral infections and viral immune evasion (109). Similarly, cell-based assays with JCV antigen stimulation elicited attenuated CD4 + T cell activation and CD8 + cytotoxicity, and an anti-inflammatory cytokine profile with lower IL-2 and elevated IL-10 cytokine levels in PML patients compared to controls (110). Although not individually diagnostic, these emerging biomarkers show promise as adjuvant testing to support early identification of PML and may 1 day become incorporated into clinical practice.

## Conclusion

Early recognition and diagnosis of PML require clinical familiarity with this rare disease among neurologists. Knowledge of clinical presentation, risk factors, supportive biomarkers, and the subtle yet distinctive radiographic features of PML outlined in this review can help clinicians maintain a high index of suspicion. A diagnosis of PML can be established with clinicoradiographic features and positive ultrasensitive CSF JCV PCR, or, in select cases, histopathologically with brain biopsy. As misdiagnosis and diagnostic delay are commonly reported in PML, early involvement of the neurologist is essential to build the diagnostic case for PML while excluding mimicking etiologies. Moving forward, broader clinical awareness of distinctive PML features, continued validation of emerging biomarkers and radiographic modalities, and standardizing diagnostic algorithms will be essential to reduce delays, avoid misdiagnoses, and expedite management for patients with PML.
